# THE JOURNAL OF APPLIED GERONTOLOGY: AN INTERNATIONAL PERSPECTIVE

**DOI:** 10.1093/geroni/igz038.706

**Published:** 2019-11-08

**Authors:** Julie Robison

**Affiliations:** University of Connecticut, Farmington, Connecticut, United States

## Abstract

The mission of applied gerontology is to bridge science and practice to benefit the health and well-being of older persons, their families, their communities, and other contexts. This presentation will provide insights from the Journal of Applied Gerontology and its attempts to publish and disseminate scholarship that has international application. Following an overview of the growing internationalization of peer-reviewed submissions to the Journal of Applied Gerontology on a variety of topics and from a range of perspectives, the presentation will highlight key achievements as well as ongoing concerns and opportunities to better achieve the goals of applying gerontological scholarship to aging contexts worldwide. Concluding comments will examine how outlets for dissemination and authors themselves can better position their work to enhance their influence on aging in an international context.

